# Mechanical and Dielectric Properties of Fly Ash Geopolymer/Sugarcane Bagasse Ash Composites

**DOI:** 10.3390/polym14061140

**Published:** 2022-03-12

**Authors:** Nattapong Chuewangkam, Theeranuch Nachaithong, Narong Chanlek, Prasit Thongbai, Supree Pinitsoontorn

**Affiliations:** 1 Department of Physics, Faculty of Science, Khon Kaen University, Khon Kaen 40002, Thailand; nattapong.ch@kkumail.com (N.C.); theeranuch.tn@gmail.com (T.N.); pthongbai@kku.ac.th (P.T.); 2 Synchrotron Light Research Institute (Public Organization), 111 University Avenue, Muang District, Nakhon Ratchasima 30000, Thailand; narong@slri.or.th; 3 Institute of Nanomaterials Research and Innovation for Energy (IN-RIE), Khon Kaen University, Khon Kaen 40002, Thailand

**Keywords:** geopolymer, fly ash, sugarcane bagasse ash, mechanical properties, dielectric properties

## Abstract

Fly ash (FA) and sugarcane bagasse ash (SCBA) are the wastes from lignite power plants and sugar industries, usually disposed of as landfills. In this research, these wastes were effectively utilized as a construction material, namely geopolymer. The effect of the SCBA (0–40 wt.%) addition to the FA geopolymers was investigated. The compressive strength of the FA geopolymers was reduced with the SCBA addition. The reduction was mainly due to the presence of the highly stable and non-reactive quartz (SiO_2_) phase in SCBA. The SCBA was not dissolved in the alkaline activated solution and hence did not contribute to the geopolymerization process. The unreacted SCBA particles remained in the geopolymer matrix but did not provide strength. However, if the amount of SCBA was about 10 wt.% or less, the impact on the characteristics and properties of FA geopolymers was minimal. Furthermore, this research also studied the dielectric properties of the FA geopolymer/SCBA composites. The relatively large dielectric constant (*ε*′ = 3.6 × 10^3^) was found for the pristine geopolymer. The addition of SCBA decreased the *ε*′ slightly due to high carbon content in SCBA. Nevertheless, the variation in *ε*′ was mainly controlled by the geopolymerization process to form the aluminosilicate gel structure.

## 1. Introduction

The Mae Moh power plant in Lampang province is Thailand’s largest lignite electricity generating station. The process uses 45 thousand tons of lignite per day, or 16 million tons per year, and emits 4.4 million tons of fly ash (FA) per year [1]. On the other hand, in the sugar industry, sugarcane is crushed to extract the juice. The fibrous residue, called bagasse, is used as a fuel source for feeding a boiler. Sugarcane bagasse ash (SCBA) is thus a residue obtained from the burning of bagasse in the sugar industry [2]. Thailand is the world’s fourth-largest sugar producer and the second-largest exporter. In 2019, the sugar production capacity was 14.58 million tons, which used sugarcane of 125 million tons, consequently producing 800,000 tons of SCBA [3]. In general, both FA and SCBA are usually disposed of as landfills. Thus, vast space is necessary to dispose of these wastes, which generate major environmental issues, harming flora, animals, and people’s health. Recycling and reusing waste should be seen from both ecological and economic standpoints. Therefore, several research studies have investigated waste utilization for various technological purposes [4,5,6]. For example, FA has been utilized in construction as a geopolymer-based material due to its high silica and alumina, low cost, and is highly reactive for geopolymerization.

Geopolymer is an inorganic binding material, mainly composed of alumina (Al_2_O_3_) and silica (SiO_2_). Polymeric structures of Al–O–Si form the fundamental building blocks of a geopolymer. Many types of materials can be used as raw materials in geopolymer production. Most of them are recycled or by-product materials, such as FA, rice husk ash, or metakaolin [7]. Alkali metal salts and/or hydroxide are usually required for dissolving silica and alumina from raw materials [8]. Generally, geopolymer provides excellent mechanical and thermal properties, high durability, high initial strength, and environmental greenness [9,10]. Geopolymer manufacturing emits 80% less CO_2_ than OPC production processes.

On the other hand, the SCBA contains a large amount of silica (62%) and some Al_2_O_3_, CaO, Fe_2_O_3_, and K_2_O. Loss of ignition (LOI) of about 10% implies the high content of unburnt organic matter [11]. Many researchers have used SCBA as pozzolanic materials, a broad class of SiO_2_ and Al_2_O_3_ materials possessing cementitious properties. SCBA was initially utilized in construction materials in 1998 [12]. The research studied the reaction between limestone and SCBA with pozzolanic characteristics by analyzing the mechanical properties of hardened cement pastes. It was found that adding a suitable amount of SCBA could enhance the compressive strength of the cement pastes [12]. According to most research, SCBA of 5–15 wt.% could be added to cement paste, mortar, and concrete to enhance its strength [5,13,14]. However, when a higher amount of SCBA was added, the mechanical properties dropped because of insufficient cement to bind aggregates and the presence of incompletely burned sugarcane bagasse and amorphous carbon in SCBA with poor strength [11,13,14,15]. However, Bahurudeen et al. reported that sieving SCBA before mixing with cement concrete increased the strength due to the complete removal of incompletely burned sugarcane bagasse and amorphous carbon [5]. As a result, SCBA could be explored as pozzolanic materials in geopolymer for improving mechanical properties.

Moreover, while SCBA could improve the strength of construction materials, it could act as a dielectric material due to its high silica content. A dielectric material is an electrical insulator that can be polarized by an applied electric field. When an electric field is applied to a dielectric material, electric charges do not flow through it as they do in an electrical conductor. Instead, they move slightly from their normal equilibrium positions, resulting in dielectric polarization [16]. Recently, many studies have been carried out on the dielectric properties of geopolymer [17,18,19,20,21]. Geopolymer is a cross-link long-chain inorganic polymer of AlO_4_ and SiO_4_ so that it requires charge balancing from alkali cations, such as Na^+^ and K^+^. The dielectric constant (*ε*′) of the geopolymer paste and mortar at 24 h after mixing were found as 3.5 and 7–10, respectively [22,23]. At room temperature, the most important variables influencing the electrical conductivity and dielectric property of geopolymers are water molecules and hydroxide [24].

In this research, we investigated the effect of SCBA addition in FA-based geopolymer paste. The mechanical strengths, dielectric properties, microstructure, and functional groups of the geopolymer/SCBA composites were studied and discussed. We demonstrated that the waste from a lignite power plant (FA) and the waste from a sugar industry (SCBA) could be turned into useful construction materials. However, the addition of SCBA had a strong effect on the microstructure and properties of FA geopolymer. Therefore, the added amount of SCBA should not be too high. Our research suggested that the SCBA concentration should be limited to about 10 wt.% of the total FA + SCBA weight. By appropriately controlling the FA and SCBA, this research has the innovation to utilize industrial wastes into more value-added products. The geopolymer/SCBA composites in this work can be used as pre-formed bricks with dielectric properties that make them very useful as functional and smart construction materials.

## 2. Experimental Section/Methods

### 2.1. Raw Materials

The source material for geopolymer preparation was high calcium lignite fly ash (FA) from the Mae Moh power plant in Lampang Province, Thailand. Sugarcane bagasse ash (SCBA) was donated from the sugar plant in Thailand (Thai Roong Ruang Research & Development Co., Ltd., Uthai Thani, Thailand). The alkaline-activated solutions were sodium hydroxide (NaOH) and sodium silicate (Na_2_SiO_3_) solutions. The 10 M NaOH solution was prepared by diluting NaOH flake (98% purity, AGC Chemicals (Thailand Co., Ltd., Samut Prakan, Thailand) in DI water. The Na_2_SiO_3_ solution was purchased from Eastern Silicate Co., Ltd., Chonburi, Thailand (12.53 wt.% Na_2_O, 30.24 wt.% SiO_2_, and 57.23 wt.% H_2_O). The mole ratio of the Na_2_SiO_3_ solution was 2.49.

### 2.2. Sample Preparation

**Figure 1:**
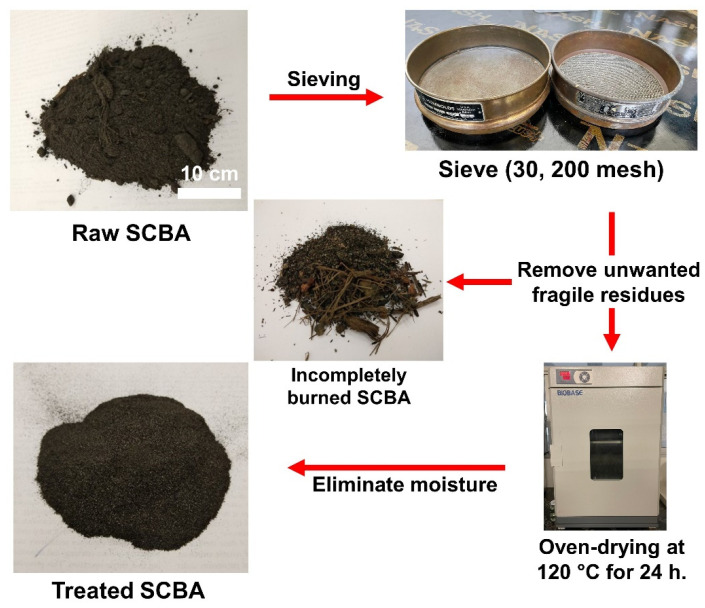
A process for treatment of the raw sugarcane bagasse ash (SCBA).

Process of treated SCBA as shown in Figure 1: the as-received SCBA was sieved to 75–600 µm (200–30 mesh) to remove unwanted fragile residues, such as incompletely burned sugarcane bagasse and other impurities. It was further treated by oven drying at 120 °C for 24 h to eliminate moisture. To prepare the FA-based geopolymer/SCBA composite, firstly, FA and SCBA were blended into a bowl for 5 min. The SCBA content was set to be 0, 10, 20, 30, and 40 wt.% of the powders’ total weight. Then, the NaOH solution was added to the mixture and stirred by a mechanical blender at 285 rpm for 5 min. Subsequently, the Na_2_SiO_3_ solution was added and mixed for another 5 min at the same speed. The ratio of Na_2_SiO_3_ to NaOH was 1.0, and a liquid to ash ratio (L/A) of 0.45 was used. After that, the geopolymer paste was poured into a 2.5 × 2.5 × 2.5 cm^3^ acrylic mold for the mechanical property test. It was also poured into a disk-shaped mold (2.4 cm diameter and 0.6 cm thick) for the dielectric properties measurement. Finally, the geopolymer paste was left at the ambient temperature for 1 h, before being wrapped with a plastic film and cured in an oven at 60 °C for 24 h. This way of curing is useful for the pre-formed geopolymer bricks but may not be suitable for manufacturing at the construction sites. The sample was kept in a control room (25 °C and 50% RH) before the tests at 7 and 28 days.

### 2.3. Characterization Techniques

The rheological behavior of the geopolymer/SCBA paste right after mixing was tested using a miniature slump (mini-slump) cone test. [25,26]. The paste was injected into a truncated conical mold, which was then lifted to a vertical position in accordance with ASTM C143. The mixture was allowed to spread after the conical mold was removed, and the diameters of the paste were measured. The workability (%*W*) was calculated as:

(1) $$\%W=\left(\frac{d-d_{0}}{d_{0}}\right)\times 100\;$$

where *d* is the spread-out diameter of geopolymer paste, and *d*_0_ is the original diameter.

X-ray fluorescence spectroscopy (XRF, Rigaku, ZSX Primus, Texas, USA) was used to evaluate the chemical composition of the as-received FA ash and the treated SCBA. The phase and crystal structure of the raw materials and geopolymer composites were examined by X-ray diffraction (XRD, Panalytical, Empyrean, Worcester, UK). Scanning electron microscopy (SEM, SEC, and SNE-4500M) was used to assess the microstructure of the geopolymer/SCBA composite. The fractured specimen after the mechanical test was gold-coated before being subjected to SEM investigation. The functional groups of the geopolymer were identified using a Fourier transform infrared spectroscope (FTIR, Bruker, TENSOR27, Boston, MA, USA). Moreover, the surface composition of the as-received FA ash and the treated SCBA were measured using X-ray photoelectron spectroscopy (XPS, PHI5000 VersaProbe II, ULVAC-PHI), at the SUT-NANOTEC- SLRI research facility, Synchrotron Light Research Institute (SLRI), Thailand.

The compressive strength was conducted on the geopolymer/SCBA specimens cured for 7 and 28 days using a universal testing equipment (Chun yen, CY-6040A12). Compressive load was applied at a rate of 50 kN/min until the specimen fractured, according to ASTM C109/C109M-20b [27]. Six samples were used for each experiment, and the average value was calculated for the compressive strength.

For dielectric measurement, the disk-shaped sample was used. The silver paste was painted at the top and bottom surfaces of the samples (diameter of 2.4 cm), before heating in air at 60 °C for 15 min to make good electrode contact. The impedance analyzer (Keysight E4990A) was used for dielectric measurement over the frequency range from 40 to 10^7^ Hz using an oscillation voltage of 0.5 V at room temperature. The relative permittivity or dielectric constant (*ε*′) was calculated from:

(2) $$\varepsilon^{\prime }=\frac{Ct}{\varepsilon_{0}A}\;$$

where *C* is the sample’s capacitance, *t* is the sample’s thickness, *ε*_0_ is the permittivity of free space (8.854 × 10^−12^ F/m), and *A* is the electrode area.

## 3. Results and Discussion

**Figure 2:**
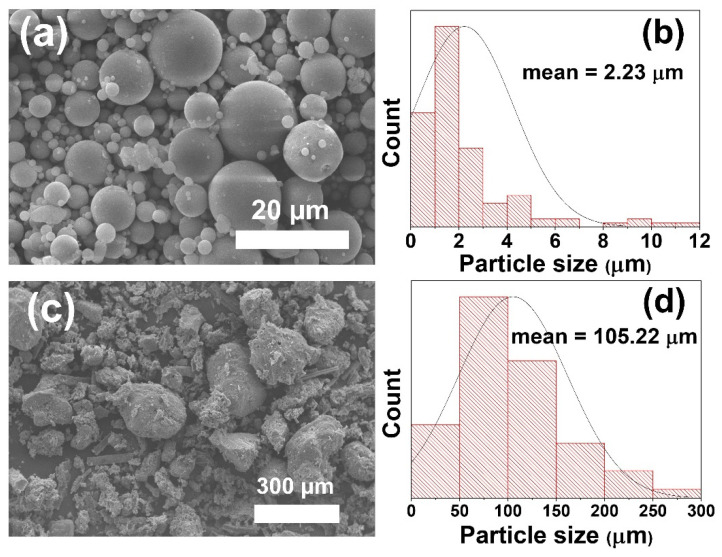
SEM micrographs and particle size distribution curves for (**a**,**b**) raw FA, and (**c**,**d**) treated SCBA.

The raw SCBA was treated, as explained in Section 2, before mixing with FA and other chemicals to form a geopolymer. The chemical compositions of the FA and treated SCBA were determined using XRF, as shown in Table 1. The major oxides of FA are SiO_2_, CaO, Al_2_O_3_, and Fe_2_O_3_. The high CaO content indicates the FA as Class C fly ash. In contrast, the main composition of SCBA is SiO_2_ (66.9 wt.%) with relatively low other oxides. The microstructure of raw FA and treated SCBA was observed under scanning electron microscopy (SEM), as shown in Figure 2, along with the particle size distribution curves. The FA was spherical particles with a smooth surface. The particle size was around 2–20 µm, with the average particle size of 2.23 μm. On the other hand, the treated SCBA was of irregular shape with a rough surface. The size of the SCBA particle was more than an order of magnitude larger than the FA particle, with the average particle size of 105.22 μm.

**Figure 3:**
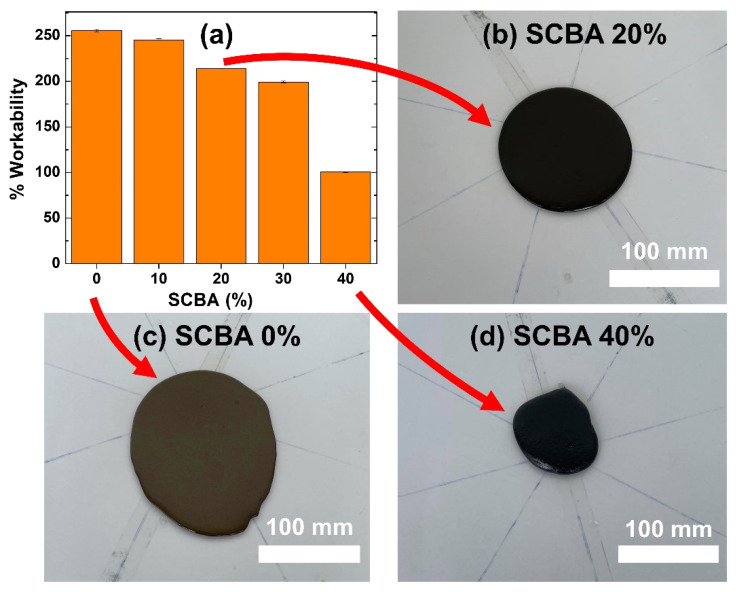
(**a**) Workability of the geopolymer/SCBA pastes; (**b**–**d**) the spread of geopolymer pastes during workability tests.

The workability of the geopolymer composite as a function of SCBA concentration is presented in Figure 3. Workability significantly deteriorates at the SCBA levels greater than 10 wt.%. Figure 3b–d show the pictures of the geopolymer pastes during the workability test. For the geopolymer paste without SCBA, the paste spreads readily and exhibits good rheological flow. However, adding SCBA into the paste increases its viscosity, especially the geopolymer/SCBA-40 wt.% paste, which becomes so viscous that it hardly flows. This result could be due to the SCBA’s high porousness and high rate of solution absorption. Workability between 150–250% is suitable for the casting and drying processes [28,29]. As a result, the optimal concentration of the added in SCBA in the geopolymer paste was between 10 to 30 wt.%.

**Figure 4:**
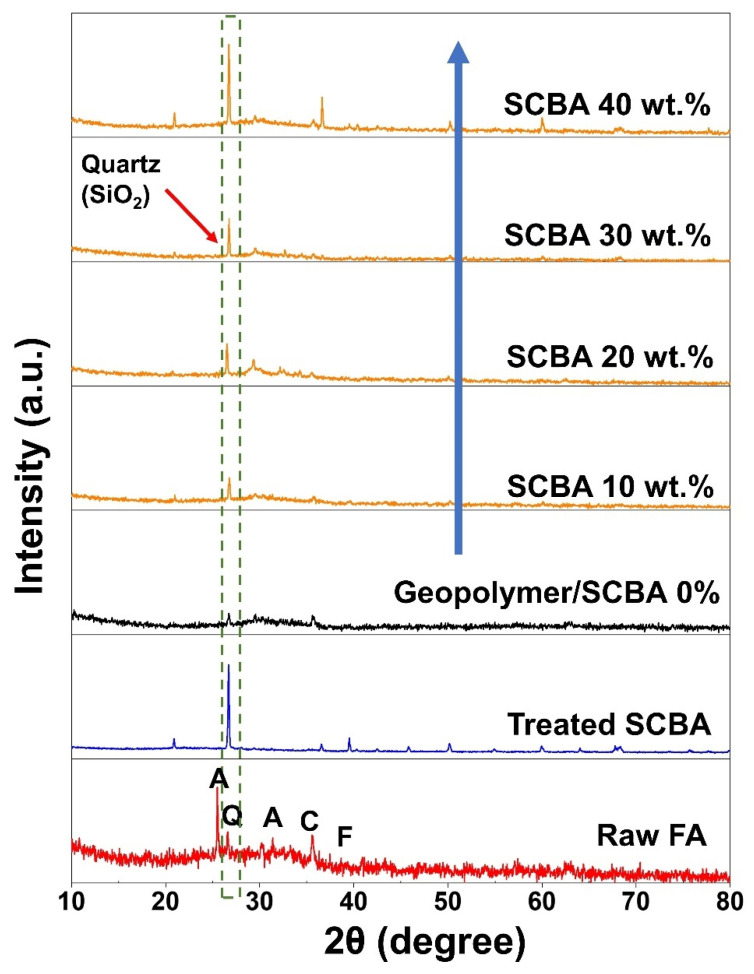
XRD patterns of the raw fly ash, treated SCBA, geopolymer paste, and geopolymer/SCBA composite. Denote the initial of phases: Q = quartz, A = anhydrite, C = calcium oxide, F = hematite.

The X-ray diffraction (XRD) patterns of raw FA, treated SCBA, geopolymer paste, and geopolymer/SCBA composite are shown in Figure 4. The raw FA shows a broad XRD hump around 2θ = 20–40°, a common characteristic of an amorphous phase. Apart from that, other crystalline peaks are observed for the raw FA, namely Anhydrite (A), quartz (Q), hematite (F), and calcium oxide (C), as indicated in Figure 4. For the treated SCBA, the XRD pattern clearly shows the high crystalline peak of the quartz phase, similar to previous reports [30]. The FA geopolymer exhibits mostly an amorphous phase with minor quartz peaks. The amorphous phase is the characteristic of geopolymeric gel as observed elsewhere [17], whereas the minor quartz peaks are due to the quartz crystal in FA. When SCBA was added to the geopolymer, the XRD pattern became the combination between the FA geopolymer and the raw SCBA. In other words, it shows the feature of the broad hump from the amorphous phase of geopolymeric gel and also the sharp quartz peak from SCBA. The intensity of the quartz peak increased proportionally with the amount of SCBA addition. It implies that the highly crystalline quartz phase of SCBA did not interact or form chemical bonds with geopolymeric gel during the geopolymerization process [31]. Thus, the SCBA did not contribute to the aluminosilicate building block of geopolymer.

**Figure 5:**
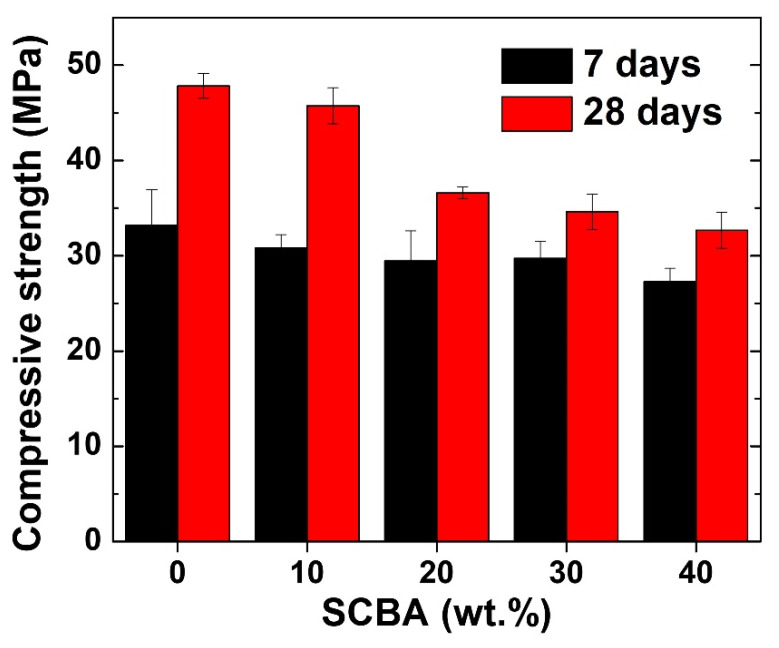
Compressive strength of geopolymer paste and geopolymer/SCBA composite pastes.

The compressive strengths of the geopolymer/SCBA composite pastes at 7 and 28 days are presented in Figure 5. The obvious point from the figure is that the strength at 28 days is much higher than at 7 days. In addition, the general trend shows that adding SCBA reduces the strength of geopolymer both at 7 and 28 days. The explanation for both observations is as follows. Geopolymerization consists of three stages: deconstruction, polymerization, and stabilization [32]. Deconstruction dissolves alumina and silica from FA in the alkaline-activated solution. The alumina and silica then form aluminosilicate geopolymeric gel during the polymerization stage. For stabilization, the gels are interconnected to form more extensive networks, and the strength of the geopolymer paste develops. However, the stabilization is slow and requires many days for the strength to be fully developed [33]. That is why the overall strengths are lower for the 7-day sample.

The reduced strength with higher SCBA could be due to the highly crystalline quartz (SiO_2_) in SCBA. Although the basis of the geopolymer structure is an Al-O-Si polymeric chain [34], the initial alumina and silica sources need to be firstly dissolved in an alkaline activated solution before proceeding to the subsequent geopolymerization stages. Thus, they are better in an amorphous form for ease of dissolution. This is what happens to the raw FA for their geopolymerization. However, since SCBA contains a significant amount of quartz, which is very stable and nearly undissolved in the alkaline activated solution, the deconstruction step was not achieved, and the geopolymerization never occurred. Therefore, SCBA did not contribute to the formation of aluminosilicate gels, as evidenced from XRD in Figure 4. Adding more SCBA means there is less FA geopolymer, which provides the strength. The other reason is attributed to the different sizes of the raw FA and SCBA, as shown in Figure 2. As the size of SCBA particles is more than 10 times larger than the FA, the SCBA is less reactive and thus had a negative impact on the geopolymer strength.

Furthermore, the effect of strength reduction with SCBA is more prominent for the geopolymer pastes aged for 28 days. The strength at 28 days reduced from 47.8 MPa for the geopolymer paste without SCBA to 32.7 MPa for the geopolymer/SCBA-40 wt.% composite, which accounts for >30% in strength reduction. On the other hand, the strength of the geopolymer/SCBA-10 wt.% composite is 45.7 MPa, almost unchanged compared to the pristine paste. Thus, it can be concluded that a small amount of SCBA (up to 10 wt.%) can be added in FA geopolymer, as an effective way for utilizing SCBA wastes, without sacrificing its mechanical property.

**Figure 6:**
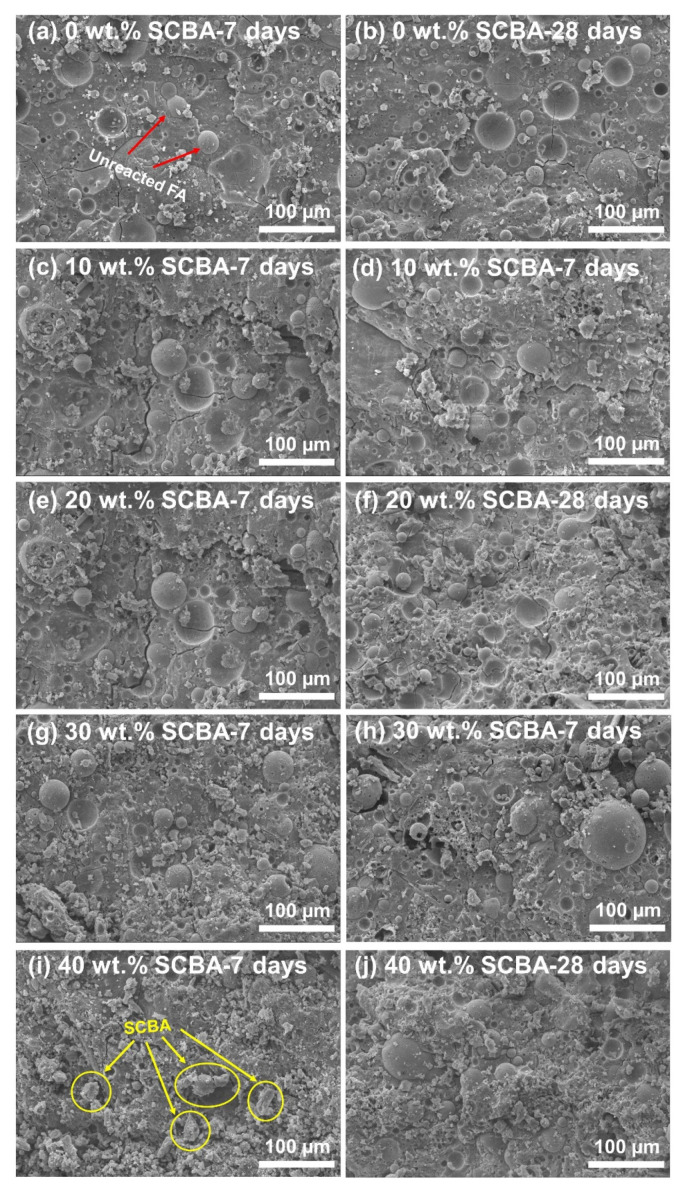
SEM micrographs of the geopolymer pastes at 7 and 28 days with SCBA of (**a**,**b**) 0 wt.%, (**c**,**d**) 10 wt.%, (**e**,**f**) 20 wt.%, (**g**,**h**) 30 wt.%, and (**i**,**j**) 40 wt.%.

The microstructures of geopolymer/SCBA pastes were examined, as illustrated in Figure 6. The fractured surfaces were examined after mechanical tests at 7 and 28 days. The distinct features from the SEM images are that the geopolymer paste without SCBA is fully dense with a smooth surface due to the formation of aluminosilicate gels from geopolymerization. However, few unreacted FA particles could still be observed (red arrows). In contrast, the surface of the composite pastes is rougher and is covered by flaky unreacted particles. These particles are partly unreacted FA but mostly are unreacted SCBA, which mainly consists of stable crystalline SiO_2_. As more SCBA was added to the geopolymer composites, a higher fraction of unreacted SCBA particles is observed (yellow circles in Figure 6i). It was reported that the number of unreacted particles and the contact between them and the geopolymer matrix had a substantial negative impact on the overall strength of the material [35]. Thus, the observation from SEM also supports the changes in compressive strengths. As more SCBA particles were added to the geopolymer composite, these SCBA particles did not involve geopolymerization. Instead, it resulted in more unreacted particles left in the geopolymer matrix, which led to decreased mechanical property.

**Figure 7:**
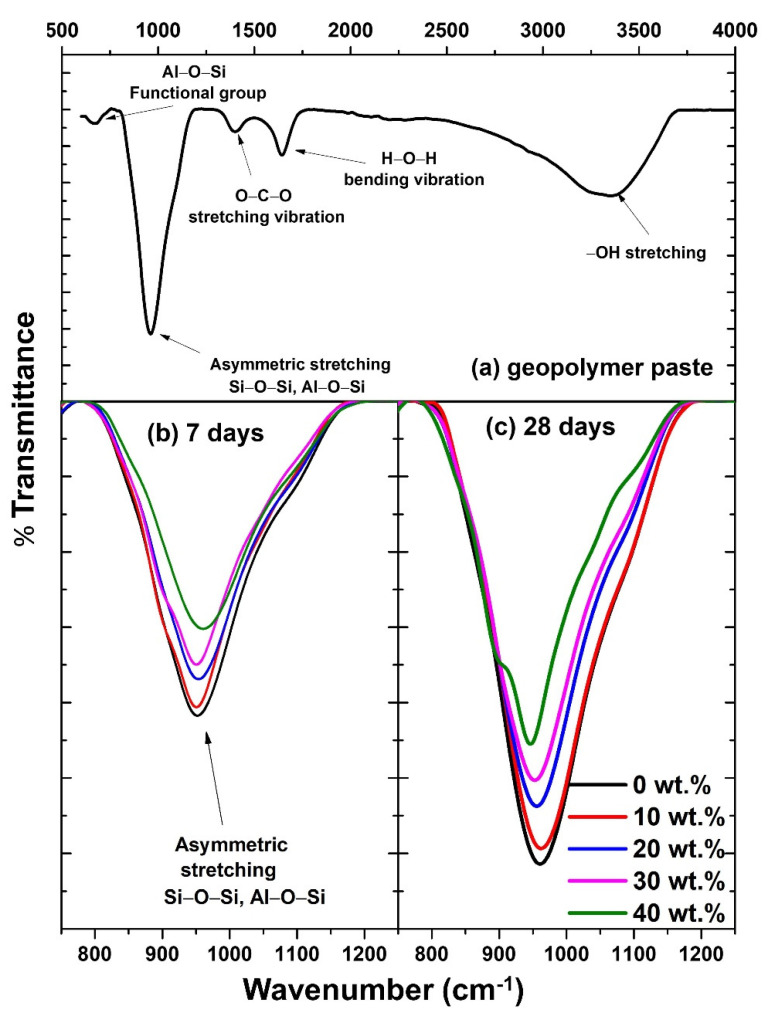
FTIR spectra of the geopolymer/SCBA composite pastes: (**a**) wide scan from 500 to 4000 cm^−1^; (**b**,**c**) expanded views from 850 to 1250 cm^−1^ for determining the change in Si-O-Si and Al-O-Si band as a function of SCBA wt.%.

It should be noted that the SEM images in Figure 6 are not significantly different between the 7-day and 28-day. This implies that the microstructure of the geopolymer paste did not change much by aging. However, the geopolymerization process still goes on at the chemical bonding level. To prove this, the FTIR spectra were measured, as shown in Figure 7. Figure 7a shows a broad scan spectrum of the geopolymer paste. Several absorption bands are observed, for instance, the symmetric stretching of Al-O at 684 cm^−1^ [36], the asymmetric stretching vibrations of Si-O-Si or Al-O-Si at 948 cm^−1^, [36,37], and the stretching vibration of O-C-O at 1416 cm^−1^ [38]. Moreover, water absorption on the geopolymer surface resulted in the bending vibration of H-O-H at 1648 cm^−1^ [39], and the hydroxyl (–OH) functional groups at 3356 cm^−1^ [40].

Geopolymerzation is related mainly to the asymmetric stretching vibrations of Si-O-Si or Al-O-Si at 948 cm^−1^, as they indicate the formation of aluminosilicate building blocks. Therefore, we expanded that band spectra and compared them amongst different samples, as shown in Figure 7b,c. The intensity of the Si-O-Si (Al) band decreases as higher SCBA (0–40 wt.%) is added in the geopolymer composite. The same trend is observed for the samples aged 7 and 28 days. This indicates that the addition of SCBA suppresses the formation of aluminosilicate geopolymeric gels. Thus, the FTIR result is another key evidence to support the changes in mechanical properties.

**Figure 8:**
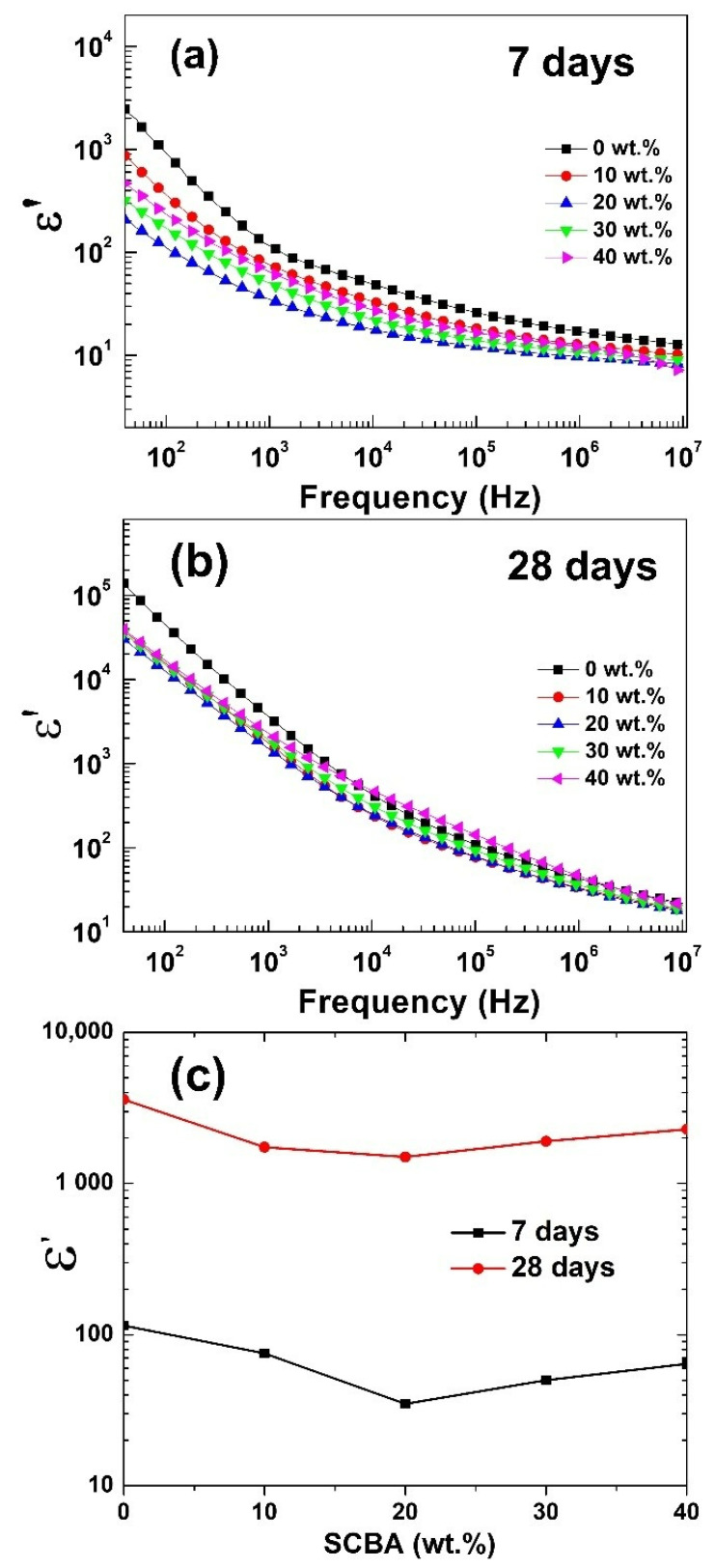
The dielectric constant *ε*′ of geopolymer/SCBA pastes at (**a**) 7 days and (**b**) 28 days. (**c**) The variation of *ε*′ at 1 kHz as a function of SCBA wt.% in geopolymer.

The dielectric properties of the geopolymer/SCBA composites were measured, as shown in Figure 8. The dielectric constants (*ε*′) for all samples decrease with increasing frequency. The dielectric response in a low-frequency region is usually caused by the interfacial polarization of composite materials or sample–electrode contact [41,42]. At low frequencies, the molecules are given sufficient time to spin and orient themselves in the direction of the applied AC at low frequencies [43]. However, at high frequencies, the time for re-orientation is not sufficient, resulting in the decreased *ε*′ from the relaxation of a polarization process within the system [44].

Furthermore, compared between the samples aged 7 and 28 days, the *ε*′ constants of 28-day geopolymers are one order of magnitude larger. For example, the *ε*′ values at 1 kHz of the geopolymer paste without SCBA were 1.2 × 10^2^ and 3.6 × 10^3^ for a 7-day and 28-day age, respectively. The increased *ε*′ with the geopolymer age could be attributed to aluminosilicate gel and relative humidity. As mentioned earlier, geopolymerization is a slow process, and the aluminosilicate geopolymeric structure continues to develop with time. Thus, the extensive network of geopolymeric gel is more developed at 28 days, leading to larger *ε*′, in an agreement in previous reports [45]. Moreover, the dielectric property is strongly affected by humidity in the samples—the higher the humidity, the lower *ε*′ [17]. Therefore, as the aluminosilicate structure is in the more advanced stage at 28 days, the relative humidity in the geopolymer composite is decreased, contributing to the higher *ε*′.

**Figure 9:**
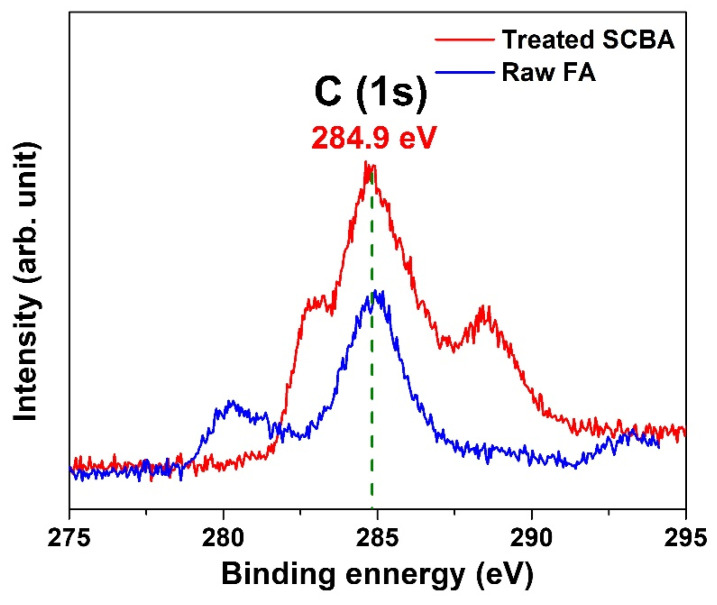
The carbon (1S) XPS spectra of the raw FA and the treated SCBA powders.

The dielectric constants were also affected by the addition of SCBA. The *ε*′ values decreased with SCBA inclusion in the FA geopolymer at 7 days (Figure 8a) and 28 days (Figure 8b). The variation of *ε*′ at 1 kHz as a function of SCBA wt.% in geopolymer is plotted in Figure 8c. The decreased *ε*′ could be attributed to an electrically conductive phase presented in SCBA. Table 2 shows the chemical compositions of the raw FA and the treated SCBA determined by X-ray photoelectron spectroscopy (XPS) analysis. Obviously, the carbon content of the SCBA is significantly higher than that of the FA. Figure 9 compares the XPS carbon peaks between FA and SCBA. It shows that the intensity of the carbon for SCBA is much higher. As carbon is a conductive material, the higher carbon fraction in SCBA reduces the sample’s insulative property and suppresses the dielectric constants. However, the effect of SCBA addition was minor when compared to the geopolymer curing age (Figure 8c). In other words, the geopolymer/SCBA composites at 28 days still exhibit much higher *ε*′ with respect to the pristine geopolymer paste at 7 days. It infers that a crucial factor in controlling the dielectric constant is the geopolymerization process to form the aluminosilicate gel structure.

## 4. Conclusions

In this research, we have utilized the waste from a lignite power plant (FA) and the waste from a sugar industry (SCBA) to fabricate a construction material, the geopolymer. The FA geopolymer without SCBA shows excellent properties, such as good rheological flow of the paste after mixing (workability = 255%), high compressive strength (47.8 MPa), and relatively large dielectric constant (*ε*′ = 3.6 × 10^3^). These excellent properties were due to the formation of the aluminosilicate gels from the geopolymerization process of FA when dissolved in an alkaline-activated solution. The amorphous phase of geopolymeric gel was detected by XRD. The SEM images showed the fully dense samples with a smooth surface of aluminosilicate gels. Moreover, the intensity of the asymmetric stretching vibrations of the Si-O-Si (Al) observed from FTIR was very strong, supporting the formation of aluminosilicate building blocks.

On the contrary, the FA geopolymer composited with SCBA showed inferior characteristics. The workability, compressive strength, and dielectric properties deteriorated as compared to the pristine geopolymer. The main reason is due to the highly crystalline quartz (SiO_2_) phase in SCBA, which is very stable and not reactive. Thus, the SCBA did not dissolve in the alkaline-activated solution and did not take part in the geopolymerization process. This led to the unreacted SCBA particles leftover in the geopolymer paste. These particles did not provide strength to the geopolymer and thus led to decreased mechanical properties. Furthermore, the high carbon content in SCBA contributed to an electrically conductive phase in the geopolymer composite, which in turn reduced the dielectric constant. However, our results suggested that if the amount of SCBA was about 10 wt.% or less, the impact on the characteristics and properties of FA geopolymers was minimal. Therefore, the FA with approximately 10 wt.% SCBA could be utilized to fabricate geopolymer composites.

## Figures and Tables

**Table 1:** Chemical composition of as-received FA and treated SCBA.

Oxide Compound	Fly Ash (wt.%)	Treated SCBA (wt.%)
Silicon dioxide (SiO_2_)	28.54	66.91
Calcium oxide (CaO)	26.37	9.48
Aluminum oxide (Al_2_O_3_)	14.94	6.66
Ferric oxide (Fe_2_O_3_)	18.14	8.11
Sulfur trioxide (SO_3_)	4.56	0.32
Potassium oxide (K_2_O)	2.80	3.49
Magnesium oxide (MgO)	2.01	1.62
Sodium oxide (Na_2_O)	1.05	0.40

**Table 2:** Chemical compositions of raw FA and treated SCBA determined by XPS analysis.

Compound	Fly Ash (at.%)	SCBA Treated (at.%)
C 1s	34.26	41.14
O 1s	52.66	46.75
Si 2p	7.92	10.88
Ca 2p	5.16	1.23

## Data Availability

The data and the code that support the results within this paper and other findings of this study are available from the corresponding author upon reasonable request.

## Abbreviations

DI	Deionization
FA	Fly ash
FTIR	Fourier transform infrared
LOI	Loss of ignition
RH	Relative humidity
SCBA	Sugarcane bagasse ash
SEM	Scanning electron microscope
XRF	X-ray fluorescence
XRD	X-ray diffraction
XPS	X-ray photoelectron spectroscopy
*ε* *′*	Dielectric constant
*C*	Sample’s capacitance
*t*	Sample’s thickness
*ε*_0_	Permittivity of free space (8.854 × 10^−12^ F/m)
%*W*	Workability
*d*	Spread-out diameter of geopolymer paste
*d*_0_	Original diameter
